# Oral health indices predict individualised recall interval

**DOI:** 10.1002/cre2.319

**Published:** 2020-08-09

**Authors:** Anna Haukka, Anna Maria Heikkinen, Jari Haukka, Minna Kaila

**Affiliations:** ^1^ Dental Care, Health Services The Social Services and Health care Helsinki Finland; ^2^ Department of Public Health University of Helsinki Helsinki Finland; ^3^ Dental Care The Health Care and Social Services Vantaa Finland; ^4^ Faculty of Medicine and Health Technology Tampere University Tampere Finland; ^5^ Public Health Medicine, Department of Public Health University of Helsinki and Helsinki University Hospital Helsinki Finland

**Keywords:** dental health, dental services research, oral health, public health

## Abstract

**Objectives:**

The individualised recall interval (IRI) is part of the oral health examination. This observational, register‐based study aimed to explore how oral health indices DMFT (decayed, missing, filled teeth), DT (decayed teeth), CPI (Community Periodontal Index, maximum value of individual was used) and number of teeth are associated with IRI for adults.

**Methods:**

Oral health examination includes an assessment of all oral tissues, diagnosis, a treatment plan and assessment and a determination of the interval before the next assessment. It is called the IRI. This cross‐sectional study population included 42,533 adults (age range 18–89 years), who had visited for an oral health examination during 2009, provided by the Helsinki City Social Services and Health Care. The recall interval was categorised into an ordinal scale (0–12, 13–24, 25–36 and 37–60 months) and was modelled using a proportional odds model. ORs less than one indicated a shorter recall interval.

**Results:**

Recall interval categories in the study population were 0–12 months (n = 4,569; 11%), 13–24 months (n = 23,732; 56%), 25–36 months (n = 12,049; 28%), and 37–60 months (n = 2,183; 5%). The results of statistical models clearly showed an association between the length of recall intervals and oral health indices. In all models, higher values of DMFT, DT and CPI indicated a shorter recall interval. The number of teeth were not so relevant. The association was not influenced when different combinations of other predictors (age, gender, socioeconomic status, chronic diseases) were included in the model. The severity of periodontitis predicted a short recall interval, for example, in the Model 1, CPI maximum value 4 was OR = 0.35 (95% confidence interval 0.31–0.40).

**Conclusions:**

The oral health indices showed a clear association with the length of the IRI. Poor oral health reduced IRI. The indices provide information about the amount of oral health prevention required and are useful to health organisations.

## 1 INTRODUCTION

The main purpose of an oral health examination is to prevent oral diseases and the further progression of oral diseases, such as caries, periodontal diseases, and mucosal changes (Clarkson, Amaechi, Ngo, & Bonetti, 2009; Mettes, Bruers, van der Sanden, et al., 2005). An oral examination can also confirm that there are no problems with previous dental work (Matthews & Tabesh, 2004). The time period between oral health examinations has been called the recall interval (Riley, Worthington, Clarkson, & Beirne, 2013) and it is part of the oral health examination and oral disease prevention process. The recall interval is based on information about individual risk factors as well as treatment response and oral disease history.

During an oral health examination, it is possible to obtain information about the severity of oral diseases with the help of oral health indices. The DMFT (decayed, missing, filled teeth) index has been used in oral health epidemiology to assess dental caries (Patel, Bay, & Glick, 2010; Preshaw, 2015). The DT (decayed teeth) provides information on untreated caries (Varsio, 1999). The CPI (Community Periodontal Index) records the health and/or disease of the periodontium and provides information on treatment needs (Dye, 2012; Patel et al., 2010; World Health Organization, 2013). The number of teeth has been used as an index and to provide information on oral health (National Collaborating Centre for Acute Care (UK), 2004). Recent evidence suggests that risk assessment, including past dental caries, is the best predictor of future caries (Chaffee, Cheng, & Featherstone, 2015; National Collaborating Centre for Acute Care (UK), 2004; Powell, 1998; Sheiham & Sabbah, 2010). Regarding the health of the periodontium, the risk assessment is based on the presence of microbial dental plaque biofilms, bleeding on probing (BOP) and deep pockets (Albandar, 2002; Dye, 2012; Genco & Borgnakke, 2013; Preshaw, 2015; Van Dyke & Dave, 2005). The number of teeth can be considered as risk assessment (Twetman, Fontana, & Featherstone, 2013). Sociodemographic variables are important for caries prediction models in older adults (National Collaborating Centre for Acute Care (UK), 2004; Powell, 1998). The recall interval must be an ongoing process and should be carried out every time a patient attends for an oral health review. One of the greatest challenges is agreeing on the optimal length of recall intervals.

The discussion between scheduled or individualised recall interval (IRI) has been going on more than 40 years. The aim of scheduled recall intervals has been to diagnosis dental caries early. In 1977, Sheiham proposed that recall visits should occur more than 6 months apart (Sheiham, 1977). Recommendations were modified, based on research findings, and showed slow (2 or 3 years) progression of caries through to dentine in permanent teeth (Clarkson et al., 2009; Daly, Batchelor, Treasure, & Watt, 2013; Patel et al., 2010; Sheiham, 1977). A systematic review (Patel et al., 2010) concluded that only weak evidence exists supporting scheduled recall intervals for reducing caries incidence. A few studies have included measures of caries in deciduous and in permanent teeth, periodontal diseases and oral cancer, showing inconclusive evidence on either the length or the scheduling of recall intervals for adults or for children (Beirne, Clarkson, & Worthington, 2007; Davenport, Elley, Fry‐Smith, Taylor‐Weetman, & Taylor, 2003). However, the authors of the systematic reviews pointed out that comparing or combining results from different studies of oral health recall intervals was difficult because of varying study protocols (Beirne et al., 2007; Patel et al., 2010; Riley et al., 2013).

The recommendations on the recall interval have been based on evidence that regular attenders had better functioning teeth, and were less likely to be suffering acute symptoms or to require emergency treatments, that is, had better oral health (Beirne et al., 2007; Bullock, Boath, Lewis, Gardam, & Croft, 2001; Thomson, Williams, Broadbent, Poulton, & Locker, 2010). Data from several studies suggest that instead of scheduled intervals, the dentist should determine the IRI for each patient (Kay, 1999; National Collaborating Centre for Acute Care (UK), 2004; Patel et al., 2010), based on the patient's individual needs (Mettes et al., 2006). Many studies have shown that IRI should be based on risk assessment of oral diseases and it may be longer than 6 months (Kay, 1999; National Collaborating Centre for Acute Care (UK), 2004; Nyyssönen, 1992; Patel et al., 2010; Richards & Ameen, 2002). Research, to date, has tended to focus IRI on risk assessment of caries but not register‐based information of oral health indices.

The aim of this observational, cross‐sectional, register‐based study was to explore how oral health indices were associated with IRI for adults. We hypothesized that oral health indices can be used to determine IRI.

## 2 MATERIALS AND METHODS

In Finland, the oral health examination includes an assessment of all oral tissues, diagnosis, a treatment plan and assessment and a determination of the interval for the next assessment and any treatment, which is commonly called the individualised recall interval (IRI). The dentist should also ask about the patient's health, medication, oral hygiene methods, diet, tobacco product habits, and alcohol consumption. With help of the Finnish guidelines for caries and periodontitis the dentist can make the decision of the length of the individual recall interval (Working group set up by the Finnish Medical Society Duodecim and The Finnish Dental Society Apollonia, 2014; Working group set up by the Finnish Medical Society Duodecim and the Finnish Dental Society Apollonia, 2016). Patients who have caries lesions should have also be offered a caries control programme. The guideline for periodontitis includes the recall guideline for maintenance care.

**FIGURE 1:**
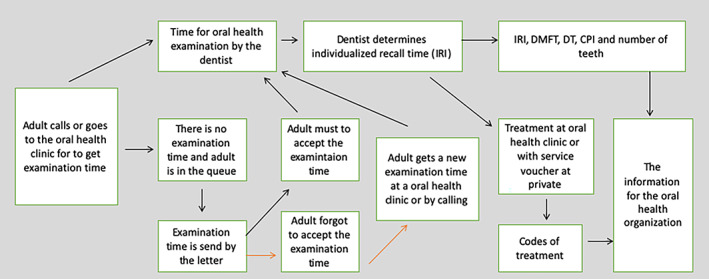
Process (from left to right) to the oral health visit in the study population and information on the indices. The patient initiates the process by asking for the appointment time for oral health examination. In Finland, all codes for treatment in municipal and private oral health clinics are provided by the board of the Finnish Institute for Health and Welfare. IRI, individualised recall interval; DMFT, decayed, missing and filled teeth; DT, decayed teeth; CPI, Community Periodontal Index

In 2009, there were 159,827 visitors (children and adults) to oral healthcare clinics run by the Helsinki City Social Services and Health Care. The adults (N = 46,461) initiated an appointment by calling or visiting the municipal oral healthcare clinic (Figure 1). If there were no available appointment times for an oral health examination, the patient's name and PIC (Personal Identification Code) were recorded in a waiting list and an appointment time was assigned after 2–3 months (Vallinkoski & Rasinen, 2012).

Our study population consisted of all adults who had visited the municipal oral healthcare clinics for oral health examinations during 2009 (January 1 to December 31, 2009), and for whom the dentist had determined an IRI at the visit. The study population consisted of N = 42,533 adults. There was no record of an IRI for 3,928 adults that were not included in the study population.

In Finland, data from different sources can be combined through the computerised register using unique PICs (Mika & Jari, 2004). The information about socioeconomic status (SES) was obtained from Statistics Finland and was categorised into eight categories, which were: self‐employed or employers, upper‐level employees, lower‐level employees, manual workers, students, pensioners, unemployed and unknown (Statistics Finland, 2020). Upper‐level employees include all those working in management tasks of public administration, enterprises or organisations, all those working in planning, research and presentation, those working in education and other employees generally with higher university degrees. Lower‐level employees include employees in management and employees in clerical, sales, care and other tasks. The information on patient's chronic diseases was accessed from special drug reimbursement held by the Finnish Social Insurance Institution (SII). The included diseases (identified by their SII codes in registry) were Diabetes mellitus (SK.103), Parkinson's disease and other comparable movement disorders (SK.110), Severe psychotic and other severe mental disorders (SK.112), Chronic cardiac insufficiency (SK.201), Disseminated connective tissue diseases, rheumatoid arthritis and comparable conditions (SK.202), Chronic asthma and similar chronic obstructive pulmonary diseases (SK.203), Chronic hypertension (SK.205), Chronic coronary heart disease and dyslipidaemia associated with chronic coronary heart disease (SK.206) and Chronic arrhythmias (SK.207). Special refunds for the cost of medicines are paid to patients who have a statement from their doctor attesting to their condition and need for medication.

Information about the oral health indices was obtained from the computerised medical records of the visit when the IRI was determined. All indices were for permanent teeth. The following indices were utilised: DMFT (Broadbent, Page, Thomson, & Poulton, 2013; Broadbent & Thomson, 2005; Reich, Lussi, & Newbrun, 1999; Sheiham & Sabbah, 2010), DT (Varsio, 1999; World Health Organization, 2013), the number of teeth (NICE National Institute for Health and Care Excellence, 2004; Reich et al., 1999) and CPI (Dye, 2012; Preshaw, 2015; World Health Organization, 2013). The CPI was recorded for the full mouth (Petersen & Ogawa, 2005; World Health Organization, 2013).

The recall interval was categorised into an ordinal scale (0–12, 13–24, 25–36 and 37–60 months). Because our independent variable (recall interval) was measured using an ordinal scale, it was modelled by a proportional odds model, which is standard for ordinal response variables (McCullagh & Nelder, 1994). The aim of modelling was to investigate a possible relationship between predictor variables such as age, gender, and oral health indices and recall interval. The purpose of modelling is to control confounding variables and find out effect of possible predictors. The results were represented as odds ratios (OR). In this study proportional odds models ORs greater than one indicated a longer recall interval than in the reference group, while ORs less than one indicate a shorter recall interval. The following variables were considered as predictors in the models: age (years), gender (male/female), SES, chronic disease indicators and oral health indices.

**FIGURE 2:**
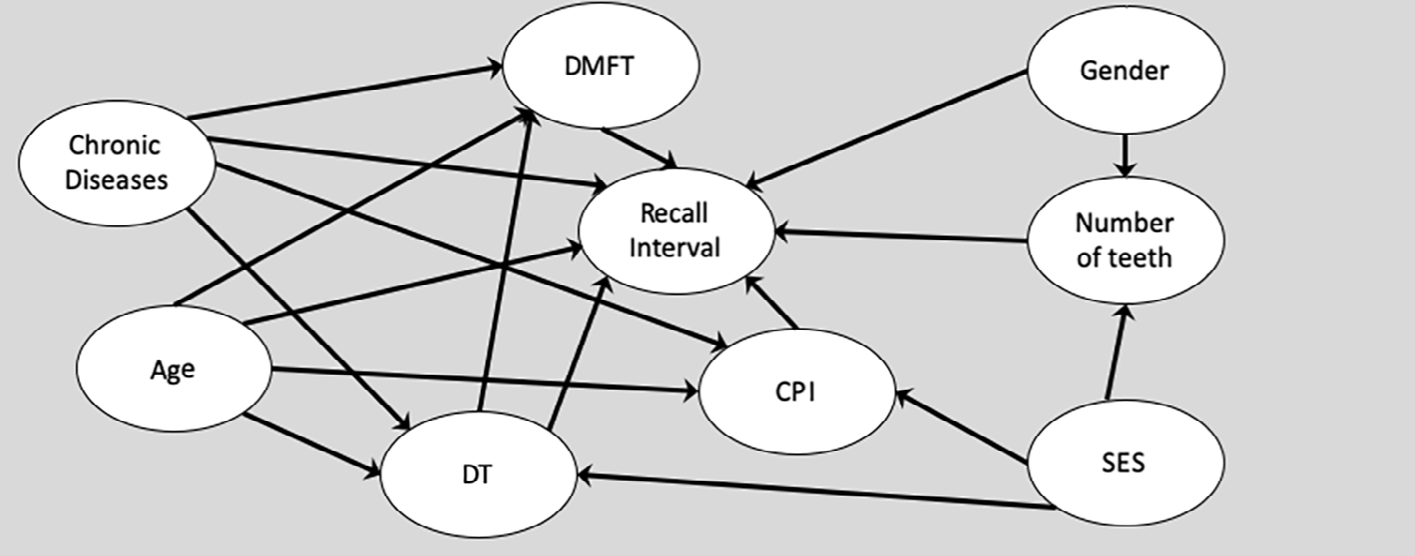
DAG‐model for recall interval. Attenders for recall interval: DMFT = decayed, missing, filled teeth, DT = decayed teeth, CPI = Community Periodontal Index, the maximum value of the individual, number of teeth, age, gender, chronic diseases (health information based the entitlement of Drug Reimbursement Register of Finnish Social Insurance Institution), SES (socioeconomic status)

Based on earlier studies, a direct acyclic graph (DAG) was created to represent the relationships between recall intervals and variables (Figure 2). Based on alternative DAG's, eight alternative statistical models were determined, in order to control for potential confounders.

The study was approved by the ethics committee of the Faculty of Medicine at the University of Helsinki (September 8, 2017 reference 09/2017); permits to use register data were obtained from the City of Helsinki (January 2018 reference 2017‐013665), Statistics Finland (January 31, 2019 reference TK‐52‐41‐19) and SII (January 31, 2019 reference 9/522/2019). We applied STROBE check list during the preparation of the manuscript.

## 3 RESULTS

The study population included 42,533 adults who had visited for an oral health examination during year 2009. There were 26,566 women (62%) and 15,967 men (38%). The IRI was determined between 0 and 60 months by the dentist at the time of the oral health examination. The IRI was recorded in months (Riley et al., 2013). In terms of recall interval categories, most of the population (n = 23,732; 56%) were given an IRI between 13 and 24 months. For the other categories, the population was divided into recall intervals of 0–12 months (n = 4,569; 11%), 25–36 months (n = 12,049; 28%), and the longest category of 37–60 months (n = 2,183; 5%) (Table 1). The distribution of socio‐economic status in the study population was very similar to that of the Helsinki city general population in 2000 (Table 2).

**TABLE 1:** Basic characteristics (demographics and oral health indicators) of the study population (N = 42,533) by recall interval determined by dentist

		Recall interval in months			
		0–12	13–24	25–36	37–60
N (%)		4,569 (11%)	23,732 (56%)	12,049 (28%)	2,183 (5%)
Age (mean, *SD*)		51.59 (19.74)	44.14 (16.82)	39.78 (14.39)	35.68 (12.21)
DMFT (mean, *SD*)		20.99 (8.17)	16.26 (8.78)	12.65 (8.72)	8.28 (7.87)
DT (mean, *SD*)		3.71 (4.22)	1.72 (2.64)	0.97 (1.75)	0.43 (1.22)
DT (%)	No	1,134 (25%)	10,374 (44%)	7,025 (58%)	1,688 (77%)
DT (%)	Yes	3,435 (75%)	13,358 (56%)	5,024 (42%)	495 (23%)
Number of teeth (mean, *SD*)		24.59 (6.89)	27.30 (5.27)	28.44 (4.58)	29.21 (4.63)
Gender (%)	Male	1907 (42%)	8,760 (37%)	4,458 (37%)	842 (39%)
	Female	2,662 (58%)	14,972 (63%)	7,591 (63%)	1,341 (61%)
CPI (%)	0	201 (4.4%)	930 (3.9%)	765 (6.3%)	142 (6.5%)
	1	214 (4.7%)	1,540 (6.5%)	888 (7.4%)	128 (5.9%)
	2	2,191 (48.0%)	15,575 (65.6%)	8,560 (71.0%)	1726 (79.1%)
	3	1,309 (28.6%)	4,434 (18.7%)	1,430 (11.9%)	138 (6.3%)
	4	620 (13.6%)	1,128 (4.8%)	333 (2.8%)	18 (0.8%)
	X (edentulous)	34 (0.7%)	125 (0.5%)	73 (0.6%)	31 (1.4%)
Diabetes (%)		326 (7.1%)	923 (3.9%)	274 (2.3%)	28 (1.3%)
Parkinson's disease (%)		34 (0.7%)	46 (0.2%)	15 (0.1%)	2 (0.1%)
Severe mental disorders (%)		250 (5.5%)	721 (3.0%)	265 (2.2%)	31 (1.4%)
Cardiac insufficiency (%)		53 (1.2%)	133 (0.6%)	29 (0.2%)	0 (0.0%)
Connective tissue diseases (%)		167 (3.7%)	434 (1.8%)	154 (1.3%)	25 (1.1%)
Chronic asthma and similar obstructive pulmonary diseases (%)		257 (5.6%)	1,017 (4.3%)	396 (3.3%)	63 (2.9%)
Chronic hypertension (%)		636 (13.9%)	1773 (7.5%)	493 (4.1%)	54 (2.5%)
Chronic coronary heart disease (%)		284 (6.2%)	657 (2.8%)	116 (1.0%)	9 (0.4%)
Chronic arrhythmias (%)		73 (1.6%)	172 (0.7%)	47 (0.4%)	5 (0.2%)
Socioeconomic status (%)	Self‐employed or employers	78 (1.7%)	626 (2.6%)	369 (3.1%)	65 (3.0%)
	Upper‐level employees	352 (7.7%)	3,868 (16.3%)	2,565 (21.3%)	592 (27.1%)
	Lower‐level employees	806 (17.6%)	6,671 (28.1%)	3,852 (32.0%)	749 (34.3%)
	Manual workers	537 (11.8%)	3,247 (13.7%)	1,664 (13.8%)	306 (14.0%)
	Students	179 (3.9%)	1,070 (4.5%)	600 (5.0%)	126 (5.8%)
	Pensioners	1940 (42.5%)	5,279 (22.2%)	1,586 (13.2%)	142 (6.5%)
	Unemployed	410 (9.0%)	1850 (7.8%)	911 (7.6%)	122 (5.6%)
	Unknown	267 (5.8%)	1,121 (4.7%)	502 (4.2%)	81 (3.7%)

*Note*: Recall interval categories: 0–12 months, 13–24 months, 25–36 months and 37–60 months. For continuous variables mean (standard deviation) are given, and for categorical variables frequencies (%). Number of teeth (wisdom teeth included). Chronic (health information based on the entitlement to the Drug Reimbursement Register of Finnish Social Insurance Institution). Socioeconomic status (Statistics Finland).

Abbreviations: CPI, Community Periodontal Index (CPI for the maximum value of individual); DMFT, decayed, missing, filled teeth; DT, decayed teeth.

**TABLE 2:** Socioeconomic status of population of City of Helsinki on year 2000 and study population year 2009

	City of Helsinki		Study population	
Socioeconomic status	N	Percentage (%)	N	Percentage (%)
Self‐employed or employers	19,959	3.6	1,138	2.7
Upper‐level employees	107,627	19.4	7,377	17.3
Lower‐level employees	134,477	24.2	12,078	28.4
Manual workers	90,422	16.3	5,754	13.5
Students	35,830	6.5	1975	4.6
Pensioners	104,886	18.9	8,947	21.0
Other (includes unemployed)	31,924	5.7	3,293	7.7
Unknown	30,349	5.5	1971	4.6
All	555,474	100.0	42,533	100.0

*Note*: Socioeconomic status was obtained from Statistics Finland and used without modification.

The mean age of the population was 43 years (aged range 18–89 years). There were 263 edentulous individuals, which was 0.6% of the study population. The periodontium was healthy (CPI = 0) in 2038 (5%) adults (Table 3). There were 20,221 (48%) adults free from baseline caries (Table 3).

**TABLE 3:** CPI (CPI = community periodontal index), maximum value of individual was used: 0 = healthy, 1 = bleeding on probing, 2 = calculus, 3 = pocket depth 4–5 mm, 4 = pocket depth 6 mm or more, X = edentulous; DT (decayed teeth): DT = 0 or DT > 0 and gender

Index		Males	Females	All
CPI	0	524 (3.28%)	1,514 (5.70%)	2,038 (4.79%)
	1	788 (4.94%)	1,982 (7.46%)	2,770 (6.51%)
	2	10,115 (63.35%)	17,937 (67.52%)	28,052 (65.95%)
	3	3,329 (20.85%)	3,982 (14.99%)	7,311 (17.19%)
	4	1,104 (6.91%)	995 (3.75%)	2,099 (4.93%)
	X	107 (0.67%)	156 (0.59%)	263 (0.62%)
	All	15,967 (100%)	26,566 (100%)	42,533 (100%)
DT	0	6,481 (32%)	13,740 (68%)	20,221(48%)
	> 0	9,486 (43%)	12,826 (57%)	22,312(52%)
	All	15,967 (38%)	26,566 (62%)	42,533 (100%)

The results of the eight models suggested an association between the length of recall intervals and oral health indices (Table 4). Higher values of DMFT, DT and CPI indicated shorter recall intervals. The association was not influenced when different combinations of other predictors were included in the model. Age was not significantly associated with the length of recall interval. However, chronic diseases were associated with shorter recall intervals. In some models, SES was associated with the length of recall interval. For women, the recall intervals were slightly shorter than for men, except in the models which included number of teeth or chronic diseases.

**TABLE 4:** Odds ratios with 95% confidence intervals from proportional odds models for ordinal scheduled intervals (0–12, 13–24, 25–36 and over 36 months and max 60 months) as a dependent variable

	Model 1	Model 2	Model 3	Model 4	Model 5	Model 6	Model 7	Model 8
CPI								
0	(Reference)	(Reference)	(Reference)	(Reference)	(Reference)	(Reference)	(Reference)	(Reference)
1	0.79 (0.71–0.89)	0.78 (0.70–0.87)	0.79 (0.71–0.89)				0.79 (0.71–0.89)	
2	0.79 (0.72–0.86)	0.78 (0.71–0.85)	0.79 (0.72–0.86)				0.79 (0.72–0.86)	
3	0.50 (0.45–0.55)	0.45 (0.41–0.50)	0.50 (0.45–0.55)				0.50 (0.45–0.55)	
4	0.35 (0.31–0.40)	0.31 (0.27–0.35)	0.35 (0.31–0.40)				0.35 (0.31–0.40)	
X	4.51 (3.39–6.00)	1.94 (1.49–2.53)	0.80 (0.72–0.89)				0.80 (0.72–0.89)	
DMFT					0.95 (0.95–0.95)			
DT	0.80 (0.79–0.80)		0.80 (0.79–0.80)	0.79 (0.79–0.80)	0.82 (0.81–0.83)		0.80 (0.79–0.80)	
Number of teeth	1.04 (1.04–1.05)		1.04 (1.04–1.05)		1.01 (1.00–1.01)	1.03 (1.03–1.04)	1.04 (1.04–1.05)	1.04 (1.04–1.04)
Age (years)	0.99 (0.99–0.99)	0.99 (0.99–0.99)	0.99 (0.99–0.99)	0.98 (0.98–0.98)	1.00 (1.00–1.00)	0.99 (0.99–0.99)	0.99 (0.99–0.99)	0.98 (0.98–0.99)
Diabetes mellitus	0.80 (0.72–0.89)	0.76 (0.69–0.85)	0.80 (0.72–0.89)	0.79 (0.71–0.88)	0.79 (0.71–0.88)		0.80 (0.72–0.89)	0.77 (0.69–0.85)
Parkinson's disease and other comparable movement disorders	0.77 (0.51–1.17)	0.56 (0.37–0.85)	0.77 (0.51–1.17)	0.95 (0.62–1.46)	0.79 (0.52–1.21)		0.77 (0.51–1.17)	0.63 (0.42–0.95)
Severe psychotic and other severe mental disorders	0.72 (0.64–0.80)	0.72 (0.64–0.81)	0.72 (0.64–0.80)	0.80 (0.71–0.90)	0.73 (0.65–0.82)		0.72 (0.64–0.80)	0.62 (0.55–0.69)
Chronic cardiac insufficiency	0.82 (0.62–1.08)	0.78 (0.60–1.03)	0.82 (0.62–1.08)	0.85 (0.64–1.12)	0.77 (0.58–1.02)		0.82 (0.62–1.08)	0.79 (0.60–1.03)
Disseminated connective tissue diseases, rheumatoid arthritis and comparable conditions	0.64 (0.55–0.74)	0.65 (0.57–0.75)	0.64 (0.55–0.74)	0.66 (0.57–0.77)	0.68 (0.58–0.78)		0.64 (0.55–0.74)	0.67 (0.58–0.77)
Chronic asthma and similar chronic obstructive pulmonary diseases	0.91 (0.82–1.00)	0.91 (0.83–1.00)	0.91 (0.82–1.00)	0.92 (0.83–1.01)	0.94 (0.86–1.04)		0.91 (0.82–1.00)	0.94 (0.86–1.04)
Chronic hypertension	0.80 (0.73–0.87)	0.83 (0.76–0.90)	0.80 (0.73–0.87)	0.81 (0.74–0.88)	0.76 (0.70–0.83)		0.80 (0.73–0.87)	0.80 (0.73–0.87)
Chronic coronary heart disease and dyslipidaemia associated with chronic coronary heart disease	0.66 (0.58–0.76)	0.68 (0.60–0.78)	0.66 (0.58–0.76)	0.71 (0.62–0.81)	0.60 (0.53–0.68)		0.66 (0.58–0.76)	0.70 (0.61–0.79)
Chronic arrhythmias	0.83 (0.65–1.05)	0.86 (0.68–1.09)	0.83 (0.65–1.05)	0.91 (0.72–1.16)	0.82 (0.65–1.05)		0.83 (0.65–1.05)	0.92 (0.72–1.16)
Socioeconomic status								
Self‐employed or employers	(Reference)	(Reference)	(Reference)	(Reference)	(Reference)	(Reference)	(Reference)	(Reference)
Upper‐level employees		1.17 (1.04–1.32)		1.16 (1.03–1.31)		1.21 (1.07–1.36)		
Lower‐level employees		0.95 (0.85–1.07)		0.98 (0.87–1.10)		0.95 (0.85–1.07)		
Manual workers		0.80 (0.71–0.90)		0.88 (0.78–1.00)		0.78 (0.69–0.88)		
Students		0.76 (0.66–0.88)		0.82 (0.71–0.94)		0.75 (0.65–0.87)		
Pensioners		0.60 (0.53–0.68)		0.70 (0.61–0.79)		0.59 (0.51–0.67)		
Unemployed		0.74 (0.65–0.84)		0.85 (0.74–0.96)		0.71 (0.62–0.81)		
Unknown		0.62 (0.54–0.72)		0.75 (0.65–0.86)		0.62 (0.54–0.71)		
Gender, female vesus male	0.83 (0.80–0.86)	0.95 (0.91–0.99)	0.83 (0.65–1.05)	0.84 (0.81–0.88)	0.89 (0.85–0.92)	1.02 (0.98–1.06)	0.83 (0.80–0.86)	1.05 (1.01–1.09)

*Note*: Odds ratios greater than unity indicate a longer recall interval than the reference group, while odds ratios less than unity indicate a shorter recall interval. Number of teeth (wisdom teeth included). Chronic (health information based on the entitlement to the Drug Reimbursement Register of Finnish Social Insurance Institution). Socioeconomic status (Statistics Finland).

Abbreviations: CPI, Community Periodontal Index (CPI the maximum value of individual), (X = value for edentulous); DMFT, decayed, missing, filled teeth; DT, decayed teeth.

**FIGURE 3:**
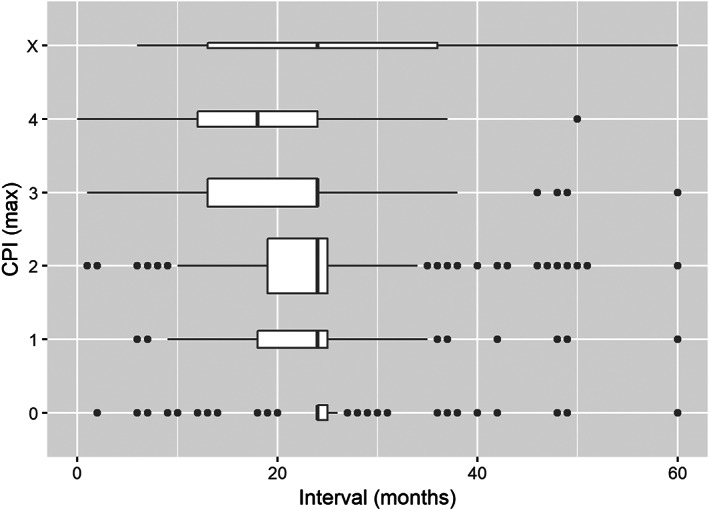
CPI (Community Periodontal Index) CPI (max) as the maximum value of individual and edentulous (X) and recall interval in months, width of the box is proportional to the size of the group, left and right end of boxes show lower and upper quintile of data, line in box indicate median, dots indicate outliers

In all models where CPI was included (Model 1, Model 2, Model 3 and Model 7), a CPI value of 4 predicted the shortest recall intervals and, for edentulous individuals, a CPI value of X (edentulous) the longest. The models with CPI indicated shorter recall intervals with the severity of periodontal disease, Model 1, CPI 4: OR = 0.35 (95% confidence interval 0.31–0.40) and Model 2, CPI 4: OR = 0.31 (0.27–0.35). The association could also be seen when comparing the severity of periodontal disease and recall interval in the study population (Figure 3). The same result was seen in models in which DT was adjusted by CPI, number of teeth, health and age (Model 3). As expected, the caries prevalence (DT) was associated with the recall interval, and higher index values reduced it, Model 3 OR = 0.80 (0.79–0.80) and Model 4 OR 0.79 (0.79–0.80).

**FIGURE 4:**
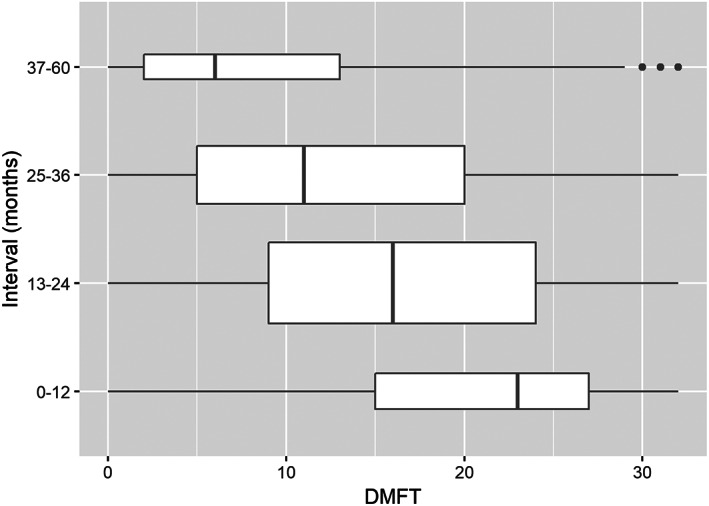
Recall interval in months and DMFT (decayed, missing, filled teeth as index), width of the box is proportional to the size of the group, left and right end of boxes show lower and upper quintile of data, line in box indicate median, dots indicate outliers

The DMFT described the previous need for treatment and baseline caries. Comparison of the DMFT and the recall interval confirmed that the higher DMFT value had association to the shorter recall interval (Figure 4). The same result was obtained in Model 5 when the DMFT was adjusted by DT, number of teeth, health and age. In general, a higher number of teeth was associated with longer recall intervals, with ORs varying between 1.01 and 1.04 in different models.

In the current study, the health information was based on information of the entitlement recorded in the Drug Reimbursement Register of SII. The presence of chronic diseases was consistent across all models except in the model of number of teeth (Model 6). In Model 8, most of the chronic diseases were associated with shorter recall intervals, especially diabetes mellitus, Parkinson's disease and other comparable movement disorders, severe psychotic and other severe mental disorders, disseminated connective tissue diseases, rheumatoid arthritis and comparable conditions, chronic hypertension, chronic coronary heart disease and dyslipidaemia associated with chronic coronary heart disease.

The SES was a potential confounder and was obtained from Statistics Finland. In three models (Model 2, Model 4 and Model 6), there were statistically significance associations between SES and recall intervals. The self‐employed or employers were the reference. Model results showed that the recall interval in upper‐level employees was longer than in other groups.

## 4 DISCUSSION

This observational, register‐based study showed a clear positive association between oral health indices and IRI for adults. We found that higher values of the DMFT and DT indices and CPI reduced the IRI, but the number of teeth were not significant regarding interval length. We also confirmed an association between chronic diseases and shorter recall intervals.

Oral health indices have previously been studied in relation to oral health recall interval in different studies, and a systematic review of DMFT and length of recall interval included 11 studies (Davenport et al., 2003). The results of included studies were conflicting or neutral (Davenport et al., 2003). In a study about the prediction of caries, using mean DMFT, a relationship between DMFT and caries progression was shown (Sheiham & Sabbah, 2010). Studies of the DT index and number of teeth were also evaluated in this systematic review (Davenport et al., 2003). The results were conflicting or neutral when comparing indices and recall intervals (National Collaborating Centre for Acute Care (UK), 2004; Davenport et al., 2003). The CPI index has mainly used in epidemiological studies (Leroy, Eaton, & Savage, 2010). In periodontal studies, outcomes have been very different and typically periodontal health has been compared between regular and irregular attenders (National Collaborating Centre for Acute Care (UK), 2004). The CPI had also been used in research exploring associations between oral health and non‐communicable diseases (Kang, Cho, & Do, 2019; Mario, Andreina, Perluigi, Giacomo, & Massimo, 2018).

The appropriate IRI is a complex decision, despite risk‐based guidelines for the recall interval (Clarkson et al., 2009). Oral disease risk varies between individuals and it is important to obtain all relevant information about general health as well as oral health before determining the IRI (Kay, 1999). Consistent with previous studies of IRI, it has been recommended to use risk‐based management in interval decisions (Fontana & Zero, 2006; Tonetti et al., 2015). However, there is not yet strong evidence for the recommendation (Beirne et al., 2007; Riley et al., 2013). The recall interval can also be based on the classification of patients into low, moderate and high risk groups (Beirne et al., 2007). Ultimately, there is still a lack of direct evidence regarding different recall strategies (Clarkson, Pitts, Bonetti, et al., 2018; Lang, Farghaly, & Ronis, 1994). Currently recommendations regarding optimal IRI vary between countries because of differing oral health organisation management and funding.

The strength of this study is that oral health indices are based on detailed clinical information about oral health and oral health procedures, with potential confounding factors based on the PIC, that also allow linking of data from these different registers. The main factors in deciding the length of the IRI are oral health and general health: periodontitis, caries and non‐communicable diseases reduce IRI. Additionally, based on combining information from registries we were able to study the association between SES and IRI even though SES is not routinely collected during an oral health examination.

However, the following limitations should be taken into account. There was no information about modifiable risk factors such as use of tobacco products, oral hygiene habits, diet and alcohol intake. Oral malignancies were not estimated in this study, even though it is important to detect changes in the oral mucosa. In Finland, a systematic examination of the oral mucosa is part of the oral health examination, and there is the guideline for persons with asymptomatic oral changes in their oral mucosa. According to this guideline, these patients should visit the dentist once a year for a systematic visual examination of their oral mucosa (Malmström et al., 2002; Working group set up by the Finnish Medical Society Duodecim and the Finnish Dental Society Apollonia, 2019).

The IRI is a decision involving both the dentist and the patient. Extensive research has shown that adults who regularly attend a general oral healthcare practice had better oral health, including less caries and fewer loose teeth and bone loss compared with adults who did not attend regularly (Bullock et al., 2001; Thomson et al., 2010). Both untreated caries and periodontitis can cause tooth loss (Farooqi, Wehler, Gibson, Jurasic, & Jones, 2015; Fontana & Zero, 2006). The CPI method as a full‐mouth record of the periodontal status should identify patients without evidence of periodontitis as well as patients with periodontitis (Preshaw, 2015). When assessing an oral health examination, the dentist should ask the patient about risk factors or indicators (e.g., use of tobacco products, diagnoses of diabetes or other chronic diseases) that could increase the probability of the occurrence of periodontitis in the future and evaluate host response factors such as age, gender and oral hygiene habits (Albandar, 2002; Krebs & Clem, 2006; Working group set up by the Finnish Medical Society Duodecim and the Finnish Dental Society Apollonia, 2016). The same questions help in the risk assessment for caries (Working group set up by the Finnish Medical Society Duodecim and The Finnish Dental Society Apollonia, 2014).

In oral health practice, we should pay attention to the IRI as a component of oral disease prevention. It is important to review the IRI at the next oral health examination. In Finland, adults are mandated to receive a dentist's appointment within 6 months of contact in non‐emergency case. Criteria for access also include a regular oral examination based on the IRI (Ministry of Social Affairs and Health, 2019). The results of previous studies have indicated that most patients prefer to make dental visits regularly (Schouten, Mettes, Weeda, & Hoogstraten, 2006; Suominen et al., 2017), and with an IRI, it is possible to equitably provide for patients (Nguyen, 2008). Patients should take part in the decision of an appropriate recall interval between oral health examinations. However, further primary research is warranted in order to assess the relative effectiveness of different recall intervals for oral health examinations.

## 5 CONCLUSION

It is suggested that oral health indices DMFT, DT, CPI and number of teeth can be used to determined IRI. The indices showed a clear positive association with the length of the IRI. In the current study we also found that indices combined with risk factors such as medical history can help the dentist in making decisions regarding IRI for patients. If there is no possibility of obtaining information about oral health risk factors, it is possible to use oral health indices in the decision for scheduling the next oral health examination. Furthermore, the indices provide information about the need for prevention of oral diseases and are useful for health organisations when planning of oral health care strategies.

## CONFLICT OF INTEREST

The authors have no conflict of interest to declare

## AUTHOR CONTRIBUTIONS

All authors have made substantial contributions to conception and design of the study. Anna Kristiina Haukka and Jari Haukka have been involved in data collection and data analysis. Anna Kristiina Haukka, Anna Maria Heikkinen, Jari Haukka, and Minna Kaila have been involved in data interpretation, drafting the manuscript and revising it critically and have given final approval of the version to be published.
